# Protrusion of the Intestines

**Published:** 1829-05

**Authors:** 


### PROTRUSION OF THE INTESTINES.

### *Sudden Protrusion of the whole of the Intestines into the Scrotum.* (WINCHESTER COUNTY HOSPITAL.)

JOHN MARSH, ætat. fifty, labourer, was brought into the hospital, having been knocked down and completely run over by a cart laden with bricks. His scrotum, on inspection, was found to be of most enormous size, extending two thirds downwards between the thighs, and measuring in circumference seventeen inches; its colour of a jet black, and its texture, from over distention, so exquisitely thin as to threaten immediate rupture from the slightest manipulation. The abdomen perfectly flaccid, and nearly empty. Immediately over the umbilicus existed a large transverse ecchymosis, indicating the exact course of the wheel over the belly. The patient was incessantly vomiting, accompanied by the most urgent retching, extremities cold, the body bedewed with profuse clammy perspirations, attended with syncope.

On being placed in bed, the viscera were returned to their natural situation without much difficulty, merely by elevating the hips, depressing the shoulders, and applying moderate and careful pressure with flannels moistened in hot poppy fomentation; the facility of reduction depending on the large opening through which the viscera had passed, together with the favorable and relaxed state of the patient. One grain of opium was now exhibited, with the view of allaying the irritability of the stomach, and the abdomen fomented, as well as the scrotum, with poppy fomentation; bottles of hot water applied to the feet.

Four o'clock P.M.—Sickness still continues unabated. Abdomen exceedingly tender, so as to confine the patient entirely to his back. Extremities warm from the application of the bottles containing hot water. Pulse feeble.

Repeat the opium pill; to use a tepid bath for ten minutes, and in one hour after a common clyster to be exhibited. The scrotum to be constantly suspended.

Second day.—Sickness has subsided. Has passed an easy night, without much sleep. Still unable to move the least in bed.

* Ibid.

Clyster has operated twice. Expresses great comfort from the bath. Pulse ninety, and feeble; skin temperate; countenance not so anxious.

Castor oil, six drachms, to be taken directly. To repeat the bath again at bedtime.

Third day.—The castor oil has operated twice copiously. Has passed a very restless night. Intense pain in the abdomen on pressure; slight tension; nausea; pulse 100, and wiry; skin dry; tongue white. The scrotum somewhat reduced in size, though perfectly black.

Thirty leeches to be applied to the abdomen directly; tepid bath as before; blister to be placed over the belly at bedtime; and to take three spoonsful of the following mixture every four hours: Sulphate of Magnesia ℥i., water, half a pint; make a mixture.

Fourth day.—Bowels have been relieved five times; the evacuations very fetid and dark. Pain on pressure nearly removed, the tension entirely. Has slept at intervals during the night. Blister has produced extensive vesication. Pulse ninety, and soft; skin perspirable; tongue white. The thighs partake of the same discoloration as the scrotum.

Effervescing saline mixture, two table spoonsful, to be taken every five hours; the bath at night; and the scrotum to be kept wet with the spirit lotion.

Sixth day.—Has continued to improve in every respect. Quite free from pain, except when endeavouring to turn in his bed. Pulse eighty-six; skin natural; tongue moist; complains of great flatulence. The lotion has had the effect of corrugating and contracting the scrotum, which is still extremely black.

Infusion of Cloves ℥i.; Aromatic Spirit of Ammonia ʒss. to be taken twice or thrice daily. Diet, a small quantity of animal food.

Twelfth day.—Quite convalescent; has been capable of sitting up in his bed for some hours, the precaution of applying a double truss having been previously taken.

He was discharged cured in three weeks from the time of the accident.

Since this patient has left the hospital, I am informed he is subject to occasional diarrhœa, which is extremely violent, and reduces him very considerably before it can be checked. He is compelled to wear his double truss both night and day, otherwise the viscera descend immediately into the scrotum in very large quantities. He was never afflicted with hernia prior to this unfortunate occurrence.*

* Ibid.

